# Non-Coding RNA-Based Biosensors for Early Detection of Liver Cancer

**DOI:** 10.3390/biomedicines9080964

**Published:** 2021-08-05

**Authors:** Sedigheh Falahi, Hossain-Ali Rafiee-Pour, Mashaalah Zarejousheghani, Parvaneh Rahimi, Yvonne Joseph

**Affiliations:** 1 Faculty of Materials Science and Materials Technology, Institute of Electronic and Sensor Materials, Technische Universität Bergakademie Freiberg, 09599 Freiberg, Germany; sedigheh.falahi@doktorand.tu-freiberg.de (S.F.); Mashaalah.Zarejousheghani@esm.tu-freiberg.de (M.Z.); Yvonne.Joseph@esm.tu-freiberg.de (Y.J.); 2 Department of Cell and Molecular Biology, Faculty of Chemistry, University of Kashan, Kashan 8731753153, Iran; rafieepour@kashanu.ac.ir; 3 Department Monitoring and Exploration Technologies, Helmholtz Centre for Environmental Research-UFZ, 04318 Leipzig, Germany

**Keywords:** liver cancer, non-coding RNAs (ncRNAs), biosensors, electrochemical, optical, electromechanical, nanomaterials

## Abstract

Primary liver cancer is an aggressive, lethal malignancy that ranks as the fourth leading cause of cancer-related death worldwide. Its 5-year mortality rate is estimated to be more than 95%. This significant low survival rate is due to poor diagnosis, which can be referred to as the lack of sufficient and early-stage detection methods. Many liver cancer-associated non-coding RNAs (ncRNAs) have been extensively examined to serve as promising biomarkers for precise diagnostics, prognostics, and the evaluation of the therapeutic progress. For the simple, rapid, and selective ncRNA detection, various nanomaterial-enhanced biosensors have been developed based on electrochemical, optical, and electromechanical detection methods. This review presents ncRNAs as the potential biomarkers for the early-stage diagnosis of liver cancer. Moreover, a comprehensive overview of recent developments in nanobiosensors for liver cancer-related ncRNA detection is provided.

## 1. Introduction

Cancer is the most common cause of death worldwide. According to the global cancer data in 2018, the universal burden of cancer has risen to 18.1 million new cases and 9.6 million cancer-related deaths. Primary liver cancer is anticipated to be the sixth most commonly diagnosed cancer and also the fourth leading cause of cancer mortality worldwide, with an estimated 841,000 new cases and 782,000 deaths, which is the highest mortality rate due to cancer [1,2]. Hepatocellular carcinoma (HCC) and intrahepatic cholangiocarcinoma (ICC) are the two major histological types of liver cancer. However, the overall liver cancer rate is greatly specified by HCC, as it comprises about 75% of all liver cancer incidences, in comparison to 15% for ICC [3,4]. Chronic infections with hepatitis B virus (HBV) and hepatitis C virus (HCV), non-alcoholic fatty liver disease (NAFLD), as well as exposure to aflatoxin, alcohol, obesity, diabetes, cirrhosis, and lipid blood are the main risk factors for liver cancer [5,6,7,8,9,10,11,12]. Unfortunately, detection of liver tumors by a physical exam is difficult, as the right rib cage covers most of the liver. Additionally, liver carcinogenesis is a long-term process and shows no specific symptoms until its later stages. Therefore, most liver tumors are detected at an advanced stage, resulting in a poor 5-year survival rate [13,14,15]. Conventional examination tools for liver tumor diagnostics are computed tomography (CT), ultrasonography (US), magnetic resonance imaging (MRI), and biopsy. These methods suffer from some limitations, such as being expensive, being operator-dependent, and having poor sensitivity, as well as requiring contrast agents to detect small tumor cells, which in most cases are accompanied by various side effects [16,17,18]. Therefore, there is an urgent need to develop sensitive, selective, relatively low-cost and simple analytical techniques for the early diagnosis of liver cancer. In the last several years, the detection and analysis of cancer biomarkers have been established as an effective cancer screening tool in diagnosis, prognosis, and treatment. Biomarkers are biomolecules in humans’ tissues and body fluids whose levels change with the development of abnormal conditions and various diseases, such as cancers [19]. Most recent research has been dedicated to identifying biomarkers, including proteins, nucleic acids, and metabolites, with the approach of enabling liver cancer diagnosis through serological testing [20,21,22].

Recently, various databases have been developed to address experimentally validated and reliable liver cancer-related biomarkers such as cBioPortal [23], BioXpress [24], OncoMX [25], and CancerLivER [26]. Accordingly, different types of biomarkers have been studied and identified in the early or late stages of HCC, including peptides, glycoproteins, enzymes, and RNAs, which can be obtained from the liver tissue and blood serum of HCC patients [21]. The most utilized pathological biomarker for HCC screening is alpha-fetoprotein (AFP) [27] which has several limitations, such as having high levels in liver cirrhosis and hepatitis, low sensitivity, and poor selectivity at an early stage of disease [28,29]. Therefore, AFP alone is not subjected to HCC detection guidelines.

The RNAs are classified into two classes: coding RNAs (cRNAs), which are translated into proteins, and non-protein coding RNAs (ncRNAs). Despite the fact that ncRNAs do not encode for proteins, they are recognized as a new and distinct class of cancer biomarkers acting as cellular regulators [30,31]. They play a remarkable role as a regulator in a variety of biological and cellular processes. Commonly, their expression levels vary in the blood serum and other body fluids of liver cancer patients [32]. ncRNAs are opening an excellent era for bringing precious diagnostic information into clinical practice and showing significant potential as an efficient biomarker for sensitive, specific, and noninvasive liver cancer detection at an early stage [33,34].

Till now, many different methods have been developed for ncRNA detection, such as northern blotting [35], microarray [36], and reverse transcriptase quantitative polymerase chain reaction (RT-qPCR) [37]. Despite the acceptable analytical performance of these techniques, they possess critical drawbacks, such as being time-consuming and requiring a large volume of sample and expensive instrumentation. Therefore, the development of new platforms is crucially required, integrating ncRNA screening in routine point-of-care diagnostics. Over the last few years, several biosensing concepts and related biosensor-based techniques have been developed to detect specific biomarkers such as ncRNAs [38,39]. Furthermore, intelligent integration of nanomaterials into biosensor structure leads to enhanced bioassay signals, sensitivity, and selectivity with higher accuracy and precision [40,41]. Electrochemical, optical, and electromechanical-based biosensors have been extensively utilized for liver cancer-related ncRNA detection to diagnose the cancer at an early stage [42].

**Figure 1:**
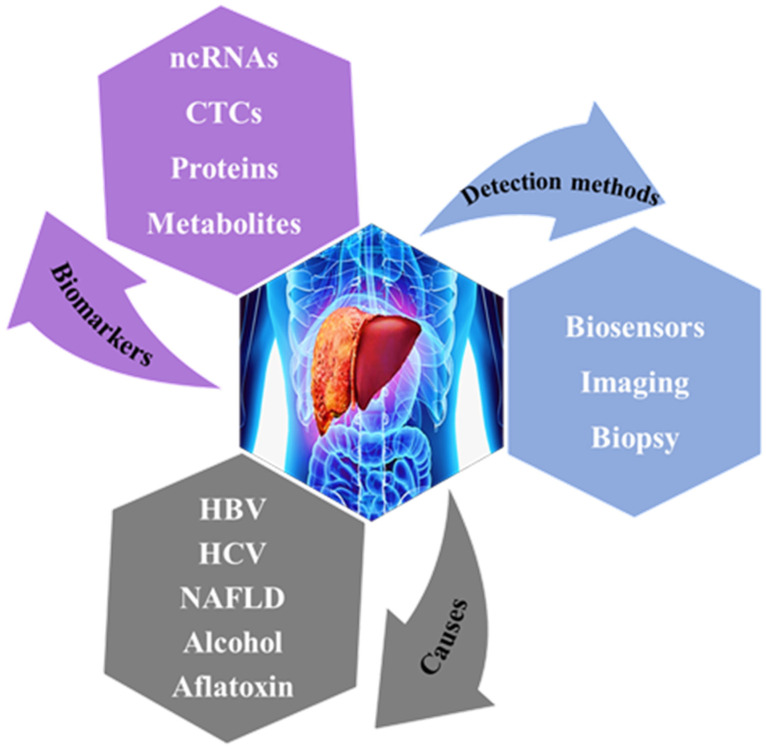
Schematic of liver cancer-associated incidence causes, biomarkers and detection methods.

In this review, we focus on ncRNAs as the potential biomarkers contributing to the major histological types of liver cancer, specifically HCC. An overview of the recent advances in the development of biosensors for liver cancer-related ncRNA detection with a special focus on electrochemical, optical, and electromechanical biosensors is provided. Further, the role of nanomaterials in the biosensing performance of the developed biosensors is highlighted. Figure 1 summarizes liver cancer-associated incidence causes, biomarkers and detection methods. 

## 2. Liver Cancer-Related Noncoding RNAs

According to the transcript length of ncRNAs, they are classified into two main classes: short ncRNAs (sncRNAs), containing less than 200 nucleotides, and long ncRNAs (lncRNAs), having more than 200 nucleotides [43], which are unable to encode proteins [44,45]. The most extensively studied sncRNA molecules (containing 18–25 nucleotides) are micro-RNAs (miRNAs), PiWi-interacting RNAs (piRNAs), small-interfering RNAs (siRNAs), ribosomal RNA (rRNA), transfer RNA (tRNA), and small nuclear RNAs (snRNAs) [46,47,48,49,50]. They play a significant role in various cellular processes and biological activities. They are involved in transcriptional and post-transcriptional regulation of protein-coding genes. Therefore, any malfunction, which affects the biogenesis pathway of sncRNAs, is strongly associated with malignant transformation, making them key contributors to tumor initiation, metastasis promotion, and disease progression. It is proven that ncRNAs have exceptional stability in clinical samples of plasma and serum which led to their rapid ascent as a novel class of biomarkers for several diseases, including cancers [51]. Among ncRNAs, most studies have been dedicated to miRNAs, as they are involved in various biological processes which underlie liver tumor formation [52]. In other words, the abnormal expression pattern of miRNA has contributed to liver cancer initiation and progression [52,53]. They may act either as oncogenes or tumor suppressors [54], can be extracted easily from tissues, plasma, serum, urine, and feces, and have a great potential as prognostic and therapeutic tools for HCC. Shi et al. validated the most HCC-associated miRNA dysregulation in a clinical setting [55]. According to their study, miR-93-5p, miR-224-5p, miR-221-3p, and miR-21-5p were up-regulated and miR-214-3p, miR-199a-3p, miR-195-5p, miR-150-5p, and miR-145-5p were down-regulated in HCC tissues. A study was performed by Zekri et al. [56] on serum miRNA panels as potential biomarkers for early detection of HCC, on top of HCV infection between HCC patients with liver cirrhosis (LC), chronic hepatitis C (CHC), and control cases. It showed that miR-122 and miR-885-5p were common miRNAs in the early detection of HCC in (LC) and control groups. According to a recent study by Yamamoto et al. [44], the accurate early-stage detection of HCC through an eight-miRNA panel comprising miR-320b, miR-663a, miR-4448, miR-4651, miR-4749-5p, miR-6724-5p, miR-6877-5p, and miR-6885-5p utilizing patient serum samples is enabled. The diagnosis of eight serum miRNAs gave >97% sensitivity and >94% specificity in the early-stage HCC detection. Some of the most relevant miRNAs which have been used for the development of early-stage liver cancer biosensors are summarized in Table 1.

Additionally, lncRNAs are significantly involved in various cellular processes such as gene transcription [57]. They have been reported to be overexpressed and proposed as biomarkers in a high number of cancers [58], as well as in liver cancer [59]. Overexpression can lead to the upregulation of lncRNAs, which raises a series of cancerous phenotypes such as elevated stemness, abnormal metabolism, and metastasis, leading to the progression of HCC. For example, lncRNA-WRAP53 in serum is an independent prognostic marker for predicting a high relapse rate in HCC patients. lncRNAs are also able to modulate the expression of non-protein-coding genes such as microRNAs. In this aspect, lncRNA H19 has been shown to inhibit HCC metastasis through activating the miR-200 family by increasing histone acetylation [60,61]. Inversely, highly up-regulated long non-coding RNA (HULC) remarkably up-regulates in liver cancer and promotes the tumorigenesis and metastasis of HCC via enhancing the epithelial–mesenchymal transition (EMT) progress in the miR-200a-3p/ZEB1 signaling pathway [62]. The exact manner is reported for linc-ROR [63], lncRNA-MUF [64], and lncRNA MALAT1 [65]. According to a study by Braconi et al., the lncRNA MEG3 and microRNA 29a can form a reciprocal regulatory loop in hepatocellular cancer [66].

**Table 1:** Sequences for miRNAs probes contributed to liver cancer.

miRNA	Sequence (5′ to 3′)	References
miRNA-122	UGGAGUGUGACAAUGGUGUUUG	[67,68,69,70]
miRNA-148b	GCCTGAGTGTATAACAGAACTT	[70]
miRNA-192	GGCTGTCAATTCATAGGTCAG	[70]
miRNA-Let7a	UGAGGUAGUAGGUUGUAUAGUU	[71,72]
miRNA-21	UAGCUUAUCAGACUGAUGUUGA	[73]
miRNA-199a	ACAGUAGUCUGCACAUUGGUUA	[74]
miRNA-223	UGUCAGUUUGUCAAAUACCCC	[75]
miRNA-125-b	UCCCUGAGACCCUAACUUGUGA	[76]

Regarding the important role of lncRNAs, they can serve as potential HCC biomarkers with high sensitivity, alone or in combination with other molecules in order to improve specificity. It is to be noted that lncRNA HULC [77] and homeobox (HOX) transcript antisense intergenic RNA (HOTAIR) [78,79] are favorable noninvasive biomarkers which have been utilized in the fabrication of biosensors for the early detection of HCC.

## 3. Other Biomarkers Related to Liver Cancer

As mentioned, the standard and the most commonly used biomarker for patients at risk of liver cancer, especially HCC, is AFP [80]. It has been shown that AFP shows a sensitivity of about 41–65%, with a specificity of 80–94% for HCC detection when a cut-off value of 20 ng/mL is used [28]. However, AFP alone is not subjected to liver cancer detection guidelines, as a high amount of AFP is also detected in the serum of patients with cirrhosis, HBV, and HCV [33]. Besides ncRNAs and AFP, a large number of serum-based proteins have been used as potential predictive biomarkers including: Des-gamma carboxyprothrombin (DCP) [81], Osteopontin [82], Midkine (MDK) [83], Dikkopf-1 (DKK1) [84], Glypican-3 (GPC-3) [85], Alpha-1 fucosidase (AFU) [86], Golgi protein-73 (GP-73) [87], and lens culinaris agglutinin-reactive fraction of alpha-fetoprotein (AFP-L3%) [88]. A recent study by Shen et al. indicates that serum DKK1 could enhance the diagnostic accuracy of HCC better than AFP [84]. According to this study, Serum DKK1 was able to distinguish HCC from chronic liver and also detect HCC in early-stage patients having normal AFP levels. However, most of these proteins have not shown superiority over AFP. To address this deficiency of protein biomarkers for liver cancer detection, a combination of markers (i.e., AFP, osteopontin, and DKK1) has been shown to make improvements in the sensitivity and specificity of HCC diagnosis [82]. Carr et al. [88] revealed that the HCC detection rate could almost be increased to 85.9% by combination detection of DCP, AFP, and AFP-L3%. In addition to serum-associated biomarkers, tissue biomarkers can function as appropriate targets for early diagnosis and development of antimetastatic vaccines/drugs. The most highlighted tissue biomarkers for HCC and ICC are Glypican-3 (GPC-3) [89], Hepatocyte paraffin 1 (Hep Par 1) [90] and Heat shock protein 70 (HSP70) [91].

In order to enhance the specificity and sensitivity of biosensors, multi-marker detection has been shown to be a powerful method for early liver cancer diagnosis. Due to the ultra-low concentration of miRNAs in patient samples, the simultaneous detection of miRNA and a protein biomarker shows great promise to achieve more sensitive and selective liver cancer diagnostic methods. In this regard, Cheng et al. [92] developed an ultrasensitive sensing strategy for liver cancer detection utilizing a combination of microRNA-223 and AFP as efficient HCC biomarkers. Remarkably, this strategy demonstrated the potential applications in clinical settings. Yu et al. [76] introduced a multi-marker diagnosis method for early HCC detection based on surface plasmon resonance (SPR) using AFP and miRNA-125b. The proposed platform showed a clinical detection range of AFP and miRNA-125b with concentrations lower than 200 pM. However, this method was unable to distinguish HCC from ICC. To address this issue, Zhu et al. [93] developed a frequency shift Raman-based sensing (SERS) method which responses three important challenges of liver cancer diagnostics: multiplex serum miRNA quantification for early-stage HCC detection; simultaneous quantification of serum miRNA and AFP in HCC patients; and quantification of serum miRNA for discrimination between HCC and ICC. They could detect miR-26a-5p, miR-223, and AFP simultaneously. They offered a cheap and accurate approach towards multiplex assaying of serum microRNAs for the early detection and discrimination of primary liver cancers. 

## 4. Biosensors for Liver Cancer-Related ncRNA Detection

The field of DNA nanotechnology has shown a remarkable impact on biomedicine, cancer research, diagnosis, and, in particular, biosensing. It has provided a versatile promising platform in the creation of novel ncRNA biosensors [94] through sub-picomolar-specific biomarker detection [95]. Regarding the key role of ncRNAs in the early detection of cancers and diseases, several DNA-based biosensing tools have been developed and reviewed [96,97,98,99]. The biosensor must have the ability to convert a specific biological recognition event into a measurable signal. In principle, biosensor systems based on nucleotide sequence base pairing measure nucleic acid hybridization events on the surface of the transducer [100], which is related to the analyte concentration. Blake et al. conducted a comprehensive review on biosensors for microRNA detection, emphasizing various sensing techniques and DNA-based biosensor principles [101]. Further, extensive efforts have been devoted to designing biosensors for the simultaneous detection of multiple biomarkers [102,103]. In a recent study, Mao et al. [104] reviewed the isothermal nucleic acid signal amplification strategies towards HCC-associated miRNA detection. Briefly, this signal amplification approach will result in a remarkable enhancement of the detection sensitivity of HCC-associated miRNAs. These studies are concerned with the possibility of applying the detection strategies in clinical practice, designing point-of-care devices for analysis of HCC-associated miRNAs, and simultaneous analysis of multiple HCC-related miRNAs or multiple groups of samples to achieve an accurate and fast diagnosis of HCC. As of yet, the most widely used DNA-based biosensors for the detection of cancer-related biomarkers use electrochemical, optical, and electromechanical (mass, surface stress, resonance) transducers depending on the type of biological response.

### 4.1. Electrochemical Biosensors

**Figure 2:**
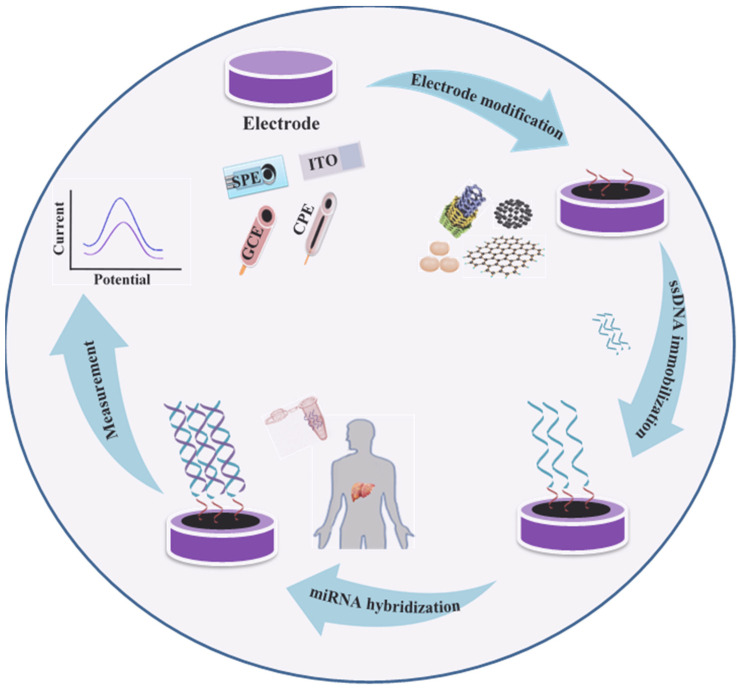
Schematic of various steps of miRNA biosensor fabrication including: surface functionalization of electrodes such as screen printed electrode (SPE), glassy carbon electrode (GCE), carbon paste electrode (CPE), or indium tin oxide electrode (ITO); immobilization of ss-DNA and further hybridization with complementary miRNA; and signal analysis through electrochemical methods (differential pulse voltammetry (DPV), cyclic voltammetry (CV), Chronoamperometry, and electrochemical impedance spectroscopy (EIS)).

Among various DNA-based biosensors, electrochemical biosensors offer an excellent promise for biomarker detection because of their attractive advantages such as simplicity, speed, low cost, and the possibility of miniaturization [105]. The basic working principle of most electrochemical DNA biosensors is depicted in Figure 2.

**Figure 3:**
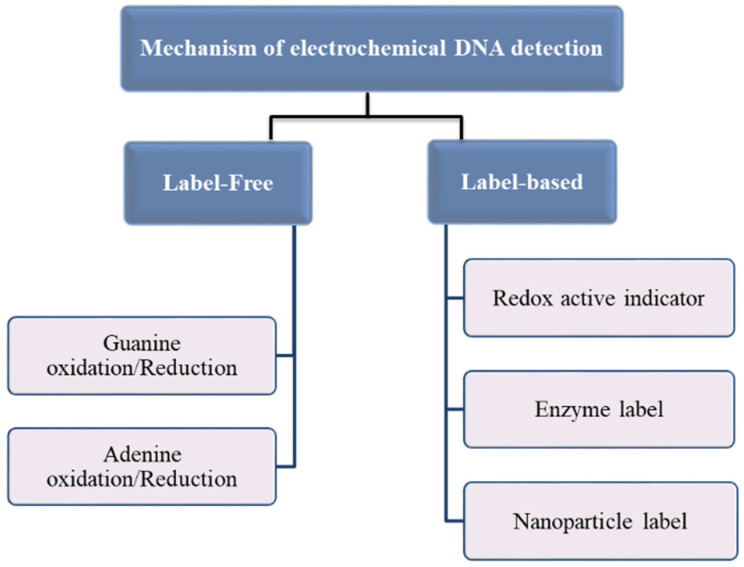
Mechanism of electrochemical DNA detection.

An important factor in the design of DNA-based biosensors is the immobilization of the nucleic acid probe onto the transducer surface, which affects the overall biosensor performances, such as sensitivity, selectivity, and reproducibility [106]. Two essential methods of DNA hybridization detection are label-free and label-based approaches (Figure 3). Most of the label-free electrochemical DNA detections are based on the changes in the redox properties of guanine and adenine in the structure of the DNA backbone. The basic principle of label-free electrochemical DNA detection is based on the interaction of guanine and adenine groups of DNA probes with its complementary thymine and cytosine bases of the target during hybridization, which can cause changes in the number of free guanine or adenine moieties available to sustain redox activities [107,108,109,110,111]. In a labeled approach, each of the capture or target probes could carry an electroactive label, for instance, an enzyme [112,113], nanoparticle [114,115,116], or an active redox indicator, such as methylene blue (MB), ferrocene (FC), etc. [38,117,118] A DNA hybridization event is detected through changes in electrochemical behavior or the redox activity of electroactive labels [106].

Advancements in nanotechnologies and the use of new nanomaterials such as carbon nanotubes (CNTs) [119], graphene (G) [120], metal nanoparticles [121], nanocomposites [122], etc. as immobilization matrices in DNA-based biosensors have enabled the design of highly sensitive and specific sensing platforms, making them attractive for the detection of ncRNA cancer biomarkers [123].

**Figure 4:**
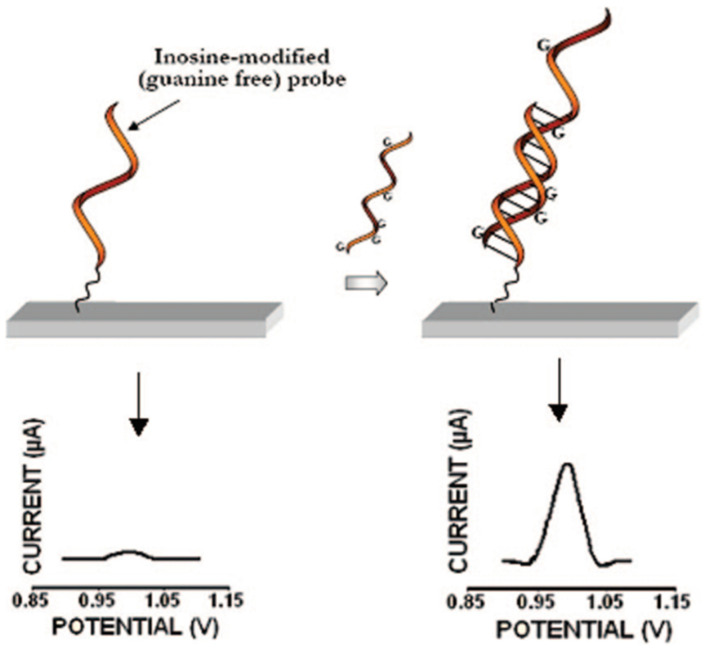
Illustration of label-free electrochemical biosensor based on guanine oxidation [124], reprinted with permission from American Chemical Society.

Recently, a great number of nanobiosensors have been developed in order to detect liver disorders at an early stage and also for making therapeutic decisions. As a reliable and valid biomarker for the early-stage diagnosis of liver cancer, many studies have been dedicated to improving the sensitive and selective detection of miRNA-122. For the first time, Lusi et al. [124] proposed a label-free electrochemical biosensor for sub-picomolar miRNA-122 detection with high specificity and a limit of detection (LOD) of 0.1 pM. The detection principle was based on guanine oxidation consequent to the formation of a hybrid between the miRNA and its inosine substitute capture probe immobilized on the surface of the screen-printed electrode (SPE). Oxidation of guanine during the hybridization event generates an electrical signal on the electrode surface, which is related to analyte concentration (Figure 4). Undeniably, this study opened a way towards tissue-specific miRNA detection. In order to apply miRNA-122 detection to the clinical level and also lower the assay time, Kilic et al. [125] designed a simple and reproducible biosensor based on G-modified pencil graphite electrodes (PGEs) for miRNA-122 detection in an RNA sample isolated from HUH-7 cell lines of HCC without any need for pre-concentration or purification. In this study, exploiting the oxidation of the guanine signal, the LOD of 1.06 pM for miRNA-122 was achieved. A significant advantage of this study was decreasing the immobilization and hybridization period to 30 min compared with previously reported biosensors [124].

Efforts for designing a sensitive electrochemical biosensor for miRNA-21 detection continued with high interest in utilizing G in different forms or composites as a platform for biological fragment immobilization. G and its related derivatives, such as graphene oxide (GO) and reduced graphene oxide (rGO), have been some of the most popular choices in the field of biosensors due to their superior electrical, mechanical, and thermal properties [126]. On the other hand, metal nanoparticles (NPs) have been widely used as powerful signal indicators and effective electrode modifiers in DNA biosensors because of their unique characteristics, including small size, high surface-to-bulk ratio, and interesting optical, electric, and catalytic properties [127]. The combination of G, GO, or rGO and metal NPs makes new ways of designing hybrid materials for electrochemical biosensing applications. In this regard, Kasturi et al. [67] proposed a highly sensitive and selective electrochemical DNA biosensor based on rGO/gold nanoparticles (AuNPs) for the detection of miRNA-122. The coated gold electrode with rGO/AuNP nanocomposites showed a remarkable capability for miRNA-122 detection with a wide linear range and enhanced sensitivity as low as 1.73 pM. Eco-friendly synthesis of the rGO/AuNP nanocomposites, superior electron transfer characteristics, as well as the large surface area could make it an efficient material for the modification of electrode surfaces.

To enhance the sensitivity of miRNA-122 detection, Wang et al. [128] fabricated a novel electrochemical miRNA biosensor based on direct growth of electroactive Prussian blue (PB) on a GO-modified DNA electrode. The PB/GO-based electrochemical sensing interface was fabricated via the assembly of GO on a DNA probe-modified gold electrode through π-stacking, followed by in situ growth of highly electroactive PB on GO through incubation in Fe^3+^ and Fe(CN)_6_^4−^ solution. Upon addition of miRNA-122 to the modified electrode, due to the weak affinity of GO with the DNA/RNA hybrid rather than a single-stranded DNA probe, the GO/PB will depart from the electrode surface. Consequently, the electrochemical response of PB at the electrode surface will be reduced, and the miRNA-122 can be monitored. The obtained well-defined electrochemical response, as well as the high sensitivity of 1.5 fM towards miRNA-122 of designed biosensor, can be attributed to the synergy of GO and PB in nano sizes. The composite of PB/GO possesses two functions, i.e., a discriminator for the probe DNA and the hybridized duplex and excellent electrochemical output response. However, a very long DNA immobilization period of 24 h is a drawback of this biosensor which does not meet the requirement for fast, real-time analysis.

In another work, in order to improve the catalytic efficiency and sensitivity and decrease the immobilization period to 3 h, a composite of Pt-Pd bimetallic nanodendrite/nanoflower-like clusters (PtPd BND/BNF) on GO/Au NPs/horseradish peroxidase (HRP) was designed for the detection of HULC in liver cancer [129]. The application of GO provides more active sites for the higher loading of PtPd BND/BNF, which results in a wide linear response to HULC in the concentration range of 0.001 to 1000 pM with an impressive LOD of 0.247 fM. Due to the catalytic characteristic of PtPd and the catalytic potentiation of the BND/BNF structure, PtPd/GO could enhance the catalytic efficiency toward H_2_O_2_ in cooperation with HRP and achieve triple-catalysis (Pt, Pd, and HRP). As efforts for screening liver cancer-related lncRNAs continued, Soda et al. [78] developed an amplification-free electrochemical biosensor for lncRNA (HOTAIR) detection with a profoundly LOD of 1.0 fM with excellent reproducibility (% RSD = < 5% for *n* = 3). 

HOTAIR sequences extracted from designated cells and plasma samples and ovarian cancer patients were magnetically purified and isolated. Applying avidin-biotin affinity, streptavidin-coupled HRP was attached to biotinylated capture probes, followed by a sandwich hybridization method in which the target HOTAIR hybridized with a screen-printed gold electrode-modified second capture-probe. This event was monitored by the amperometry technique utilizing the H_2_O_2_/HRP/hydroquinone (HQ) system. To authorize this method for analyzing HOTAIR expression levels in patient samples, the standard RT-qPCR method was employed. This highly proved assay could be used as a low-cost and reliable platform in conventional clinical frameworks for screening cancer-related lncRNAs.

**Figure 5:**
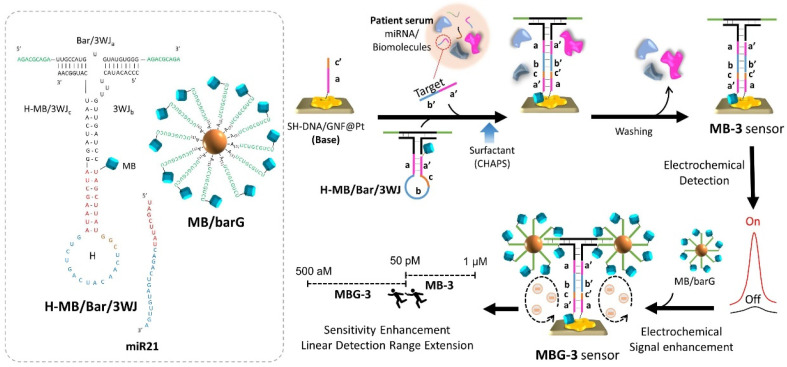
Schematic diagram of the relay-race electrochemical biosensor. Left panel: the predicted structures and nucleic acid sequences of the H-MB/Bar/3WJ, miR21 and MB/barG [73], reprinted with permission from Elsevier.

Mohammadniai et al. [73] developed a novel sensing platform based on a three-way joint (3WJ) miRNA structure, utilizing an MB-modified hairpin (H-MB) structure as one leg to function as the sensing element and the other two legs hybridized with barcode gold nanoparticles (MB/barG) as the signal amplifiers. The addition of miRNA-21 resulted in opening the hairpin moiety and further hybridization with a DNA-modified gold nanoflower/platinum electrode (GNF@Pt) to form the MB-3 sensor (Figure 5). A considerable LOD of 135 aM in a broad linear detection range from 1 μM to 500 aM for miRNA-21 as a liver cancer biomarker was reported. The amplified signal could be attributed to the electrodeposition of Pt with a GNF structure generating a significant electron conductive nanostructure with a high surface to volume ratio. Additionally, the addition of MB/barG boosted the electrochemical signal of the MB by almost 230 times (MBG-3).

Besides miRNA-122, miRNA-let 7a has also received lots of attention as a potential biomarker of liver cancer in assembling the electrochemical biosensors [72]. In this regard, a highly sensitive platform for miRNA-let 7a determination in hepatocellular carcinoma patients and hepatic cancerous cultured cell lines Huh7 and HepG2 has been developed [130]. The sensor was constructed of carbon paste (CP) modified with silver nanoparticles (AgNPs) and extracted propolis (bee glue) (AgNP/P). Utilizing electrochemical impedance spectroscopy (EIS), an LOD of 1 aM was reported in this study. To enhance the sensitivity and LOD for miRNA-let 7a detection, Elhakim et al. [71] proposed a biosensor based on a nanocomposite of chrysin, AuNPs, and CNTs. They could reduce the immobilization and hybridization time to 30 min and quantify miRNA-let 7a in the zepto-molar level (zM). Furthermore, the proposed biosensing assay was applied to determine miRNA-let 7a in serum samples of HCC patients and hepatic cancerous cultured cell lines Huh7 and HepG2 with satisfactory results. Zhang et al. [131] proposed a novel isothermal electrochemical biosensor for the sensitive detection of miRNA-221 without using nanomaterials. The biosensor was fabricated based on a combination of the target-catalyzed hairpin assembly (CHA) and super sandwich amplification strategies. Based on the dual signal amplification strategies, the proposed biosensor showed a superior selectivity and sensitivity towards miRNA-221 with a LOD of 0.6 pM. Moreover, this approach was utilized to monitor miRNA-221 in the real sample, and the results were in striking agreement with those obtained using qRT-PCR. Table 2 summarizes the specifications and LODs of various electrochemical biosensors developed for liver cancer-related ncRNA detection.

### 4.2. Optical Biosensors

**Figure 6:**
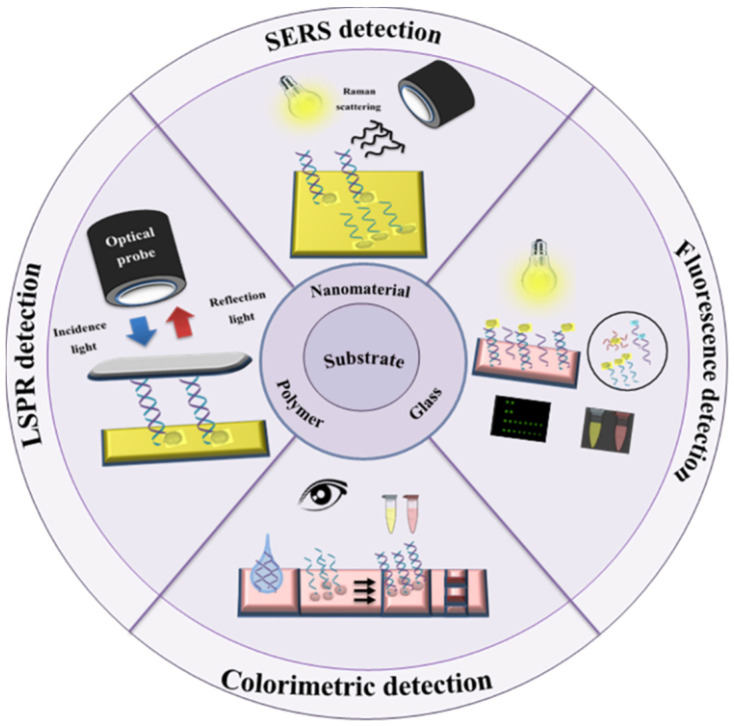
Schematic illustration of optical-based DNA biosensor sensing approaches: LSPR, SERS, colorimetric and fluorescence detection.

Optical biosensing strategies are novel classes of detection methods, which have attracted increasing attention in bimolecular analysis, especially in cancer diagnostics and therapy, as well as drug discovery technology [132,133,134,135,136]. The main advantages of optical biosensing systems include cost-effectiveness, simplicity, rapid results, and avoiding the use of radiochemical assays. Optical assays such as colorimetric, chemiluminescence, SERS, and localize surface plasmon resonance (LSPR) biosensors are the most developed systems in DNA biosensing [137,138,139,140] (Figure 6).

**Figure 7:**
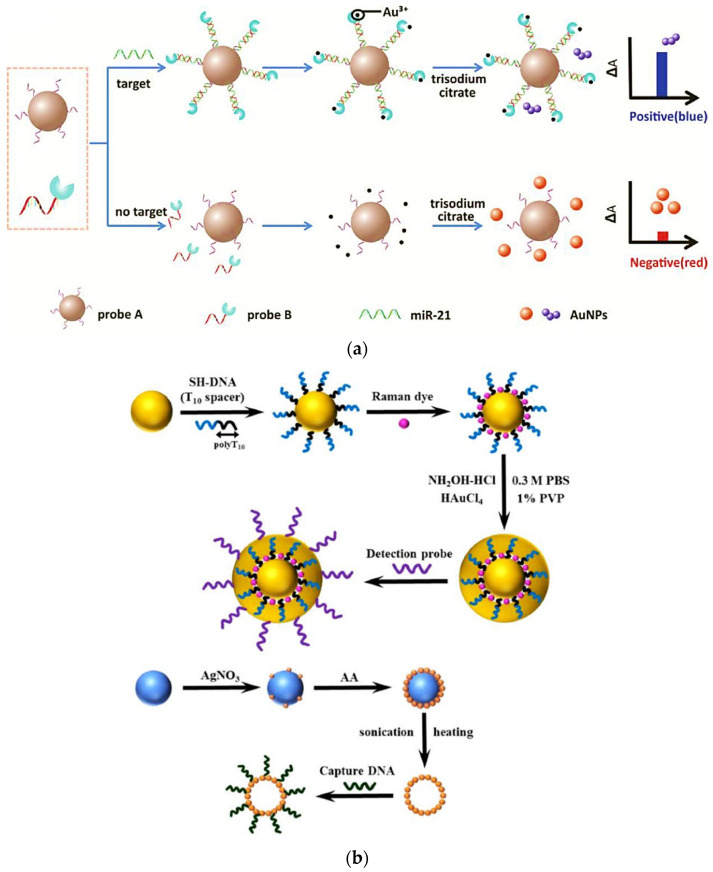
(**a**) Schematic diagram of MC colorimetric biosensor for miR-21 detection [143], and (**b**) schematic illustration of Synthetic procedures of Raman dye-coded Au-NPs using DNA-modified AuNPs as templates and the biotemplating synthesis strategy of AgHMSs using bacteria as template (blue sphere) and ascorbic acid (aa) as Ag reductant agent [75], (**a**) reprinted with permission from Elsevier, and (**b**) reprinted with permission from American Chemical Society.

Unique properties of nanomaterials such as notably small sizes, unique optical properties, a high specific surface area, and versatile surface chemistry allow special interactions with a variety of capture molecules. This enabled the development of a variety of plasmonic applications on the basis of the colorimetric sensing, which is provided by metal nanoparticles [141]. AuNPs are the mainly used nanomaterials in optical biosensors due to their unique properties [142]. The color of AuNPs, less than 15 nm in diameter, appears red and can be changed to purple or blue upon interparticle plasmon coupling [138]. Such a color change can be observed by naked eyes and also used as signal output in colorimetric biosensors for miRNA biomarkers. Considering the elegant color-forming feature of AuNPs and the strong chelating capability between EDTA•2Na and Au^3+^ metal chelator-labeled signal amplification, a notably sensitive colorimetric assay was proposed for miRNA-21 detection with a low LOD of 8.9 fM and excellent stability [143]. In this strategy, EDTA•2Na-labeled oligonucleotides operated as the plasmonic signal supraregulator probe, and oligonucleotides labeled SiO_2_ microparticles (SiO_2_MPs) were performed as detecting platforms. In the presence of target miRNA-21, the EDTA•2Na-labeled oligonucleotide probes were immobilized on the SiO_2_MPs platform through the sandwich structure. The assembled sandwich biosensor could chelate Au^3+^ to regulate the generation of AuNPs, resulting in colorimetric signals to measure the various miRNA-21 concentrations (Figure 7a).

A dye-free colorimetric assay for miRNA-122 detection was developed by Wang et al. [144] This method was based on duplex-specific nuclease (DSN)-assisted signal amplification coupled to the AuNPs in which two processes were involved. First, designation of a target-mediated probe by a DSN enzyme and probe-triggered AuNP aggregation, which acts as a switch for signal output. Second, construction of the reaction system consisting of a probe complex composed of two partly complementary DNA probes and two sets of distinct oligonucleotide-modified AuNPs with sequences complementary to a DNA probe in the probe complex. In the presence of complementary miRNA-122, the probe complex was invaded, resulting in the miRNA-probe hybridization, acting as a substrate of the DSN enzyme and releasing the other probe to link to the AuNPs. The proposed method attained a detection of miR-122 in the range of 20 pM to 1 nM and a LOD of 16 pM. Additionally, this detection assay was reported to discriminate single-base differences and successfully applied to quantify miR-122 in cancerous cell lysates accurately. 

Several types of optical biosensors have been used for ncRNA detection. Among them, the SPR biosensors have been shown to be incredibly efficient for the direct sensing of ncRNAs [142]. To enhance the sensitivity of optical biosensors, a wide variety of amplification strategies have been employed such as (i) target recycling reaction [145], (ii) magnetosome amplification method [146], and (iii) DNAzyme-based reaction [147]. These strategies helped to reduce the LOD up to sub-pM levels. However, these methods are expensive, complex, and require a longer measuring time. For instance, the standard SPR method needs less than a few minutes of operation, while the addition of an amplification strategy extends the read-out to 1 h. Sipova et al. [148] designed a rapid and label-free detection of miRNA-122 utilizing portable SPR sensor technology and a DNA/RNA antibody-based assay. They could detect the miRNA in less than 30 min at concentrations down to 2 pM, just by introducing an antibody that recognizes and binds to the DNA/RNA hybrids. To improve the diagnostic sensitivity and specificity simultaneously, Yu et al. [76] developed a multi-marker SPR-based sensor by the immobilization of anti-AFP antibodies and the DNA probes on the surface of the SPR sensor for the recognition of AFP and miRNA-125b as combined HCC markers. They increased the sensitivity of the developed sensor using the double antibody sandwich method (DASM) and S9.6 antibody enhanced method. The results indicated that the AFP detection could meet the clinical detection range (25–400 ng/mL), and the LOD of miRNA-125b reached 123 pM in serum. In another attempt, a SERS biosensor was fabricated for the simultaneous detection of multiple liver cancer-related microRNA biomarkers. A new strategy was explored for the synthesis of nanogap-based SERS nanotags by coating AuNPs with thiolated DNA and nonfluorescent small encoding molecules and a simple method for green synthesis of hollow silver microspheres (Ag-HMSs) with bacteria as templates [75]. Based on the sandwich hybridization assay, DNA-conjugated SERS nanotags as SERS nanoprobes and capture DNA-conjugated Ag-HMSs as capture substrates were used for the simultaneous quantification of the three liver cancer-related miRNAs (Figure 7b). Multiplexed assays successfully distinguished three target miRNAs with a limit of detection in the pM range.

In recent years, electrochemiluminescence (ECL) has received much attention as a promising tool for various biological sample detections due to extraordinary properties of low background, high sensitivity, and being user friendly [149]. Different types of luminescence species have been applied in ECL, such as quantum dots (QDs) [150], Ru(bpy)_3_^2+^ [151], luminol [152] graphene quantum dots (GQDs) [153], and PtPd embed GQD [154].

Benefiting from the combination of metal NPs with GQDs, Li et al. [77] proposed an ultrasensitive sandwich-type ECL sensor based on the electrodeposited AuNPs as the matrix and Au@Ag/GQDs as a signal indicator. Actually, due to the synergistic effect between Au and AgNPs, Au@Ag core-shell nanoparticles presented a large specific surface area, better catalysis, and superior electronic transmission capacity in comparison to the individual Au or Ag NPs. On the other hand, Au@Ag core-shell NPs could promote the ECL performance of GQDs, resulting in the highly sensitive detection of HULC with a wide linear range from 1 fM to 5 nM with an extreme lower LOD of 0.3 fM.

### 4.3. Electromechanical Biosensors

Electromechanical biosensors [155,156,157,158] are interesting analytical devices that use the basic principle of a response to a change in mass. They are label-free, highly sensitive, and offer non-invasive disease screening, gene tests, and diagnostics. In comparison to optical and electrochemical biosensors, electromechanical biosensors are highly sensitive to minor mass changes and are also capable of detecting molecules that don’t have electrically conducting property or optical signal [159,160]. Major sensing platforms are cantilever, quartz crystal microbalance (QCM), and surface acoustic wave (SAW) [70,161,162].

**Figure 8:**
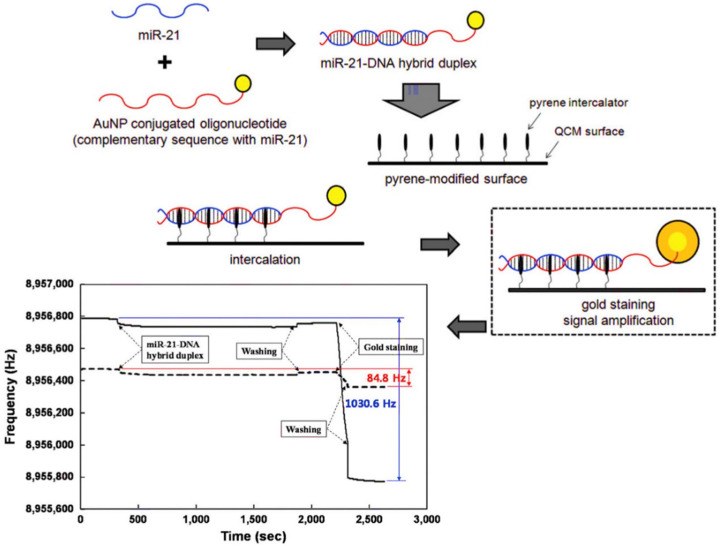
Schematic illustration of miRNA detection mechanism using QCM biosensor [165], reprinted with permission from Royal Society of Chemistry.

A biosensor based on QCM technology is one of the most popular label-free biosensing platforms for the detection and quantification of a wide range of biomolecules. Furthermore, the high sensitivity and short detection process offered by QCM biosensing assays make them attractive for the development of novel and disposable diagnostic tools [163,164,165]. QCM biosensors consist of a piezoelectric crystal (quartz) coated with a metal electrode and their function is based on the change in frequency of the quartz in response to the adherence of a target molecule. DNA probes can be immobilized on transducer surfaces via chemical interaction or electrostatic adsorption onto cationic films utilizing the negatively charged phosphate group on oligonucleotide single strands, Au–S or avidin/streptavidin-biotin bonds [166]. The addition of a sample and the hybridization of DNA/RNA causes a mass loading at the crystal surface and creates a frequency response. In a recent study, a QCM biosensor was developed for the sensitive and specific detection of miRNA-21 [165]. First, miRNA-21 was incubated with AuNP-conjugated single-stranded DNA containing the complementary sequence of miRNA-21 and then introduced to a pyrene-functionalized QCM sensor chip. Next, gold staining solution was added to the modified surface, resulting in the catalyzed deposition of metallic gold onto AuNPs captured on the chip surface (Figure 8). Utilizing this strategy, an LOD of 3.6 pM in the linear range of 2.5 pM to 2.5 μM was obtained. Furthermore, this approach was able to detect miRNA-21 in the total RNA extracted from the human brain and A549 cell line. Therefore, this assay might have potential as an alternative in clinical diagnosis. Huang et al. [167] studied the development of the QCM-based immunosensor for detecting AFP. They pointed out a high mass sensitivity (0.299 Hz/ng mL^−1^) and sensing linearity (99.23%) in a range of 10–40 ng/mL AFP concentration.

**Figure 9:**
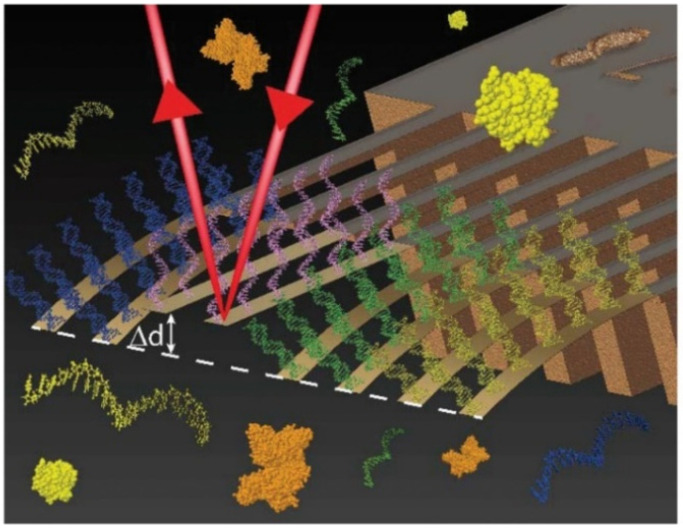
Illustration of target binding on the cantilever array surface. Perspective is from inside the fluidic chamber. The laser from the optical beam read out detection method is shown reflecting away from a cantilever surface, out of the chamber, and towards the detector. Differential deflection (Δd) arises between the in situ reference probes and target sensitive probes [70], reprinted with permission from Royal Society of Chemistry.

In the past few years, cantilever-based biosensors have been shown to be the most attractive candidate for practical application in the early diagnosis of cancers because of their desirable characteristics such as simple batch fabrication, reliability, and cost-effectiveness [155,158]. They can be operated in two different modes: static mode or dynamic mode. The binding of the analyte generates a deflection or bending in the static-mode and changes the resonant frequency in the dynamic-mode. Duffy et al. [70] reported an automatic cantilever-based biosensor for non-invasive, rapid, and personalized miRNA detection for liver cancer diagnostics (Figure 9). The proposed biosensor based on static mode was able to detect miRNA-122, miRNA-148b, and miRNA-192 relevant to liver cancer using only a few microliters of sample within one hour. Specific miRNA hybridization to the upper cantilever surface induced a physical bending of the sensor, which could be detected by controlling the position of a laser that reflects from the sensor’s surface. The proposed platform may offer a new medical tool without the need to individually extend, amplify, or label each target, allowing multi target analysis from one sample. Table 3 enlists the specifications and LOD of various optical and electromechanical biosensors developed for liver cancer-related ncRNA detection.

SAW sensing platforms are alternative devices to QCM-based bioassays with higher sensitivity and a higher operating frequency. SAW-based biosensors can detect the changes in acoustic waves which are propagated at the surface of a piezoelectric substrate during the mass loading process. Since their frequency ranges are from several hundred MHz to GHz, they can record significantly small frequency shifts as a result of exceptionally small mass loadings [168,169]. To the best of our knowledge, no SAW-based biosensor has been reported to diagnose liver cancer-related miRNA biomarkers to date. The highly sensitive SAW sensor designed for pancreatic cancer RNA biomarkers [170] can be utilized as an appropriate pattern for designing such a sensor for the early detection of liver cancer. 

A list of the abbreviations used in this review is provided in Table 4.

## 5. Conclusions

The accurate determination of specific tumor biomarkers with non-invasive or minimally invasive procedures is the most promising approach to improve the long-term survival of liver cancer patients and reduce the high incidence and mortality rate of this disease. Among different types of biomarkers used for HCC diagnosis, ncRNAs are recognized as a new and distinct class of cancer biomarkers for the early-stage detection of liver cancer. Moreover, in certain cases a combination of several ncRNAs can help to overcome the limitations such as low sensitivity and poor selectivity of other HCC biomarkers, such as AFP. Regarding the key role of ncRNAs in the early detection of cancers and related diseases, DNA-based biosensors offer a promising platform for ncRNA detection and present various advantages, such as simplicity, reliability, and high sensitivity and selectivity. Due to the low amount of ncRNAs in a real human cancerous sample at the early stage of liver cancer, it is highly demanded to improve the sensitivity of related biosensors. To address this issue, various nanomaterials have been used to increase the sensitivity and selectivity of biosensors. The ultrasensitivity and the specificity of liver cancer biosensors can also be improved by detecting multiple relevant liver cancer biomarkers simultaneously and exploiting signal amplification strategies. Among the various DNA-based biosensors for ncRNA detection, much effort has been devoted to developing electrochemical biosensors. However, despite progress, just a few studies on developing biosensing platforms for the detection of ncRNA as an early-stage biomarker for cancers, particularly HCC, have been reported. So, we envision that the integration of nanomaterials with extraordinary features into the biosensing system for multi-biomarker detection provides an early and rapid diagnosis method and offers the opportunity to fabricate disposable point-of-care devices.

## Figures and Tables

**Table 2:** Summary of introduced electrochemical biosensors for liver cancer-related ncRNA detection.

Target	Technique	Sensor Material	Electrode	IP ^1^ (h)	HP ^2^ (h)	LOD	LR ^3^	References
miRNA-122	DPV	rGO/AuNP	Gold	12	1	1.73 pM	10 pM to 10 µM	[67]
	DPV	PB/GO	Gold	24	1	1.5 fM	10 fM to 10 nM	[128]
	DPV	n.m. ^4^	SPE	1	1	1 pM	5 nM to 1 μM	[124]
	DPV	Graphene	PGE	0.5	0.5	1.06 pM	0.5 to 7 μg/ml	[125]
miRNA let7a	DPV	CNT/Chrysin/AuNPs	CPE	0.5	0.5	1.0 zM	1 zM to 11 nM	[71]
	EIS	AgNPs/P	CPE	n.a. ^5^	0.5	1 aM	1 aM to 1 μM	[130]
miRNA-21	DPV	Pt	SPE	n.a.	n.a.	135 aM	500 aM to 1 μM	[73]
miRNA-221	Amperometry	n.m.	Gold	3	Over night	0.6 pM	0 to 20 nM	[131]
lncRNA (HULC)	CV	PtPd BND/BNF@GO/Au/HRP	GCE ^6^	3	3	0.247 fM	1 pM to 1 mM	[129]
lncRNA (HOTAIR)	Amperometry	n.m.	SPE	1	2	1 fM	1 fM to 1 nM	[78]

^1^ Immobilization period, ^2^ Hybridization period, ^3^ Linear range, ^4^ Not modified, ^5^ Not available, ^6^ Glassy carbon electrode.

**Table 3:** Summary of introduced optical and electromechanical biosensors for liver cancer-related ncRNA detection.

Target	Technique	Sensor Material	LOD	LR	References
miRNA-122	SPR	Antibody-based	2 pM	10 pM to 100 pM	[148]
	Colorimetric	AuNP	16 pM	20 pM to 1 nM	[144]
miRNA-21	Colorimetric	SiO_2_MPs	8.9 fM	10 fM to 0.1 pM	[143]
miRNA-125b	SPR	Antibody-based	123 pM	8 nM to 1000 pM	[76]
miRNA-223	SERS	AuNP	>pM	10 pM to 10 nM	[75]
lncRNA (HULC)	ECL	Au@Ag/GQDs	0.3 fM	1 fM to 1 nM	[77]
miRNA-122 miRNA-148b miRNA-192	Cantilever-based	Pyrene	n.a. ^1^	0 to 1 pM	[70]
miRNA-21	QCM	Ti ^2^/Au	3.6 fM	2.5 pM to 2.5 μM	[165]

^1^ Not available, ^2^ Titanium.

**Table 4:** List of abbreviations used in this review.

Abbreviation	Explanation	Abbreviation	Explanation
HCC	Hepatocellular carcinoma	CNTs	Carbon nanotubes
ICC	Intrahepatic cholangiocarcinoma	G	Graphene
HBV	Hepatitis B virus	LOD	Limit of detection
HCV	Hepatitis C virus	LR	Linear range
NAFLD	Non-alcoholic fatty liver disease	SPE	Screen-printed electrode
CT	Computed tomography	PGEs	Pencil graphite electrode
US	Ultrasonography	GO	Graphene oxide
MRI	Magnetic resonance imaging	RGO	Reduced graphene oxide
AFP	Alpha-fetoprotein	NPs	Nanoparticles
mRNAS	Messenger RNAs	AuNPs	Gold nanoparticles
ncRNAs	Non-protein coding RNAs	PB	Prussian blue
sncRNAs	Short ncRNAs	BND/BNF	Bimetallic nanodendrites/nanoflower
piRNAs	PiWi interacting RNAs	HRP	Horseradish peroxidase
siRNAs	Small-interfering RNAs	Pt	Platinum
rRNA	Ribosomal RN	Pd	Palladium
tRNA	Transfer RNA	HQ	Hydroquinone
snRNAs	Small nuclear RNAs	3WJ	Three-way joint
lncRNAs	Long ncRNAs	H	Hairpin
miRNAs	Micro-RNAs	GNF	Gold nanoflower
RT-qPCR	Reverse transcriptase quantitative polymerase chain reaction	AgNPs	Silver nanoparticles
LC	Liver cirrhosis	P	Propolis
CHC	Chronic hepatitis C	barG	Barcode gold nanoparticles
HULC	Highly up-regulated non-coding RNA	EIS	Electrochemical impedance spectroscopy
EMT	Epithelial-mesenchymal transition	CP	Carbon paste
HOX	Homeobox	zm	Zepto-molar
HOTAIR	Transcript antisense intergenic RNA	CHA	Catalyzed hairpin assembly
DCP	Des-gamma carboxyprothrombin	LSPR	Localize surface plasmon resonance
MDK	Midkine	SiO_2_MPs	Silicon dioxide microparticles
DKK1	Dikkopf-1	DSN	Duplex-specific nuclease
GPC-3	Glypican-3	DASM	Double antibody sandwich method
AFU	Alpha-1 fucosidase	ECL	Electrochemiluminescence
GP-73	Golgi protein-73	QDs	Quantum dots
AFP-L3%	Lens culinaris agglutinin-reactive fraction of alpha-fetoprotein	GQDs	Graphene quantum dots
Hep Par 1	Hepatocyte paraffin 1	QCM	Quartz crystal microbalance
HSP70	Heat shock protein 70	SAW	Surface acoustic wave
SPR	Surface plasmon resonance	aa	Ascorbic acid
SERS	Shift Raman-based sensing	FC	Ferrocene
MB	Methylene blue		

## Data Availability

Not applicable.
